# The relationship between pubertal timing and markers of vascular and cardiac structure and function in men and women aged 60–64 years

**DOI:** 10.1038/s41598-019-47164-x

**Published:** 2019-07-30

**Authors:** Rebecca Hardy, Jane Maddock, Arjun K. Ghosh, Alun D. Hughes, Diana Kuh

**Affiliations:** 1 CLOSER, UCL Institute of Education, University College London, London, WC1H 0NU UK; 2 MRC Unit for Lifelong Health and Ageing at UCL, Institute of Cardiovascular Science, University College London, London, WC1E 7HB UK; 3 Barts Heart Centre, St Bartholomew’s Hospital, West Smithfield, London, EC1A 7BE UK

**Keywords:** Risk factors, Cardiology

## Abstract

Earlier age at menarche has been associated with higher risk of coronary heart disease, but the mechanisms underlying the association remain unclear. We assessed the relationship of pubertal timing, in both men (n = 672) and women (n = 713), with vascular (carotid intima-media thickness (cIMT), pulse wave velocity (PWV)) and cardiac (left ventricular (LV) structure and function) measures recorded at age 60–64 yrs in a British birth cohort study. Regression models found that earlier menarche was associated with higher (more adverse) LV mass, LV end diastolic volume and left atrial volume, but not with other cardiac measures, cIMT or PWV. Associations were attenuated after adjustment for either adult or childhood BMI (e.g. mean difference in LV mass per year later menarche: −4.2 g (95% CI:−7.0,−1.4) reducing to −2.2 g (95% CI:−4.7,0.4) after adjustment for adult BMI). There were no associations among men, despite those fully mature at 15 yrs having higher blood pressure than the least mature group by 10.21 mmHg (95% CI:19.45,0.98). Any effect of pubertal timing on vascular and cardiac structure and function is likely to be small and primarily confounded by pre-pubertal BMI and/or mediated through adult adiposity.

## Introduction

Understanding of the relationship between pubertal timing and development of cardiovascular risk factors may lead to improved management of disease risk. Earlier age at menarche has been associated with higher risk of cardiovascular disease (CVD) and coronary heart disease (CHD) in women in some, but not all, observational studies^1–8^. A meta-analysis of 5 studies produced an estimated risk ratio (95% confidence interval) of 1.15 (1.02,1.28) for early (<12 years) versus later menarche^7^. The cardiovascular health consequences of pubertal timing in boys are less studied^7^, but findings from the UK Biobank Study suggested that men reporting that their voice had broken at a younger age than their peers had higher risks of self-reported angina and myocardial infarction than those who reported their voice breaking around the average age^4^.

Carotid intima-media thickness (cIMT), a marker of subclinical atherosclerosis, and pulse wave velocity (PWV), a marker of arterial stiffness, have been shown to add to the Framingham risk score in the prediction of CVD^9,10^, but have been little studied in relation to pubertal timing^11–13^. There is little evidence of an association between age at menarche and cIMT in women in the few studies that do exist^11–13^. Standard CV risk factors have been more extensively investigated, although evidence of associations between age at menarche and lipids^7,11,14–16^ and blood pressure (BP)^7,11,15–18^ has been mixed with some studies exhibiting associations in the expected direction but others finding no evidence of such associations. The limited numbers of studies that have included males have also found little evidence of associations between pubertal timing and CV risk factors^14,17,19–21^. We have previously found, in a British birth cohort study, that earlier puberty was associated with higher BP at 53 years in men, but that there was no association with lipid profile^14,17^.

While most research on pubertal timing has focussed on CHD, a recent study using the Women’s Health Initiative (WHI) investigated associations of reproductive characteristics including age at menarche, with incident heart failure^22^, an increasingly common and important manifestation of CVD in older people^23^. Associations observed between later age at menarche and lower risk of heart failure in age-adjusted analyses were attenuated in fully adjusted models. Left ventricular (LV) hypertrophy and LV mass have been found to be risk factors for CVD, independent of classic CV risk factors^24^ and moderate and severe diastolic dysfunction are related to increased subsequent mortality^25^. To our knowledge, no study has investigated the relationship between pubertal timing and such measures of cardiac structure and function, as early indicators of heart failure, in a general population sample.

It has been suggested that any associations between pubertal timing and CV risk may be explained by body size^6^. Early pubertal timing has been consistently related to high body mass index (BMI) in adulthood, particularly in women^7^. However, the fact that girls with earlier menarche have higher childhood BMI^26^, means that observed associations between age at menarche and CV risk could also be partially due to confounding with childhood BMI. However, as most studies lack a measure of childhood BMI and are thus unable to adjust for childhood BMI^7^, the relative contribution of childhood and adult BMI to any relationship remains unclear.

We use data from the Medical Research Council (MRC) National Survey of Health and Development (NSHD) to investigate whether prospectively collected indicators of pubertal timing in men and women are associated with vascular measures (cIMT and PWV) and LV cardiac structure and function at age 60–64. We assess the extent to which any associations are explained by both measured childhood and adult body size (height and BMI). We also assess whether the associations between pubertal timing and BP and lipids at 53 years previously observed in NSHD persisted at age 60–64 years and examine if these classic CV risk factors further explained any associations between pubertal timing and the vascular and cardiac measures.

## Methods

The Medical Research Council (MRC) National Survey of Health and Development (NSHD) is based on a nationally representative sample of 5362 births out of all the single births to married mothers that occurred in one week in March 1946 in England, Scotland, and Wales^27^. The cohort has been followed up ever since, with a clinic visit taking place in 2006–2010 when participants were 60–64 years old^28^. Study members still alive with a known address in Britain and who had not previously withdrawn from the study (N = 2856) were invited for an assessment at one of six clinical research facilities (CRF) or to be visited by a research nurse at home. Invitations were not sent to those who had died (N = 778), were living abroad (N = 570), had previously withdrawn from the study (N = 594), or had been lost to follow-up (N = 564). Of the 2856 invited, 2229 (78%) were assessed: 1690 (59%) attended a CRF and 539 were visited at home. Of the original NSHD participants, those with lower educational attainment, lower childhood cognition and those who were lifelong smokers were less likely to participate at 60–64 years. The participating sample remained representative of British men and women of the same age in terms of sex, social class and smoking behaviour when compared with the 2011 England census and the ONS Integrated Household Survey. NSHD, however, had a higher proportion of working participants, a higher proportion of owner occupiers and a lower proportion with limiting long-term illness^29^. The analytical sample includes 1385 study members (672 males and 713 females) who have at least one valid outcome measure and information on pubertal timing.

Ethical approval was obtained from the Greater Manchester Local Research Ethics Committee and the Scotland Research Ethics Committee for the age 60–64 years data collection. Written informed consent was obtained from the study member for each component of each data collection.

### Vascular measures

Carotid intima-media thickness (cIMT), a marker of atherosclerosis, and pulse wave velocity (PWV), a marker of arterial stiffness, which are both associated with CVD, were measured at 60–64 years in the CRFs only as these measurements could not be carried out in participant’s homes. A high resolution scanner (Vivid I, GE Healthcare) with a 12 MHz probe was used to image the left and right common carotid arteries longitudinally one centimetre proximal to the carotid bifurcation following a standardized protocol^30,31^. Analysis of attained cineloops was carried out using dedicated software (Carotid Analyser, Iowa City, Iowa) which employed semi-automatic edge detection to measure cIMT. Three end-diastolic frames from each view were selected for measurement. The mean of the six lateral measures (three right and three left) are used in these analyses. All images were analysed by two trained readers in a blinded fashion.

Carotid to femoral PWV was measured, using a Vicorder device (Skidmore Medical), by placing a 10 cm wide pressure cuff at the upper right thigh and another 3 cm partial cuff directly over the right carotid artery^31^. The cuffs were inflated simultaneously to 65 mmHg for approximately 10–15 seconds. Path length was measured between the cuffs and defined as the distance between the suprasternal notch directly to the top of the femoral cuff and PWV was automatically calculated by an integral algorithm.

### Cardiac measures

Echocardiography was also only carried out in participants attending the CRFs. Echocardiographic images were obtained using GE Vivid I machines from parasternal long axis and short axis, apical 5-chamber, 4-chamber, 3-chamber, 2-chamber and aortic views along with conventional and tissue Doppler in the 4-chamber view. Image analysis was carried out using GE EchoPac software. Wall and chamber measurements were made according to American Society of Echocardiography/European Association of Echocardiography guidelines^32^. Measures of left ventricular (LV) structure used as outcomes in analyses were LV mass (LVM), LV end diastolic volume (LVEDV) and relative wall thickness (RWT) which are indicators of LV hypertrophy and remodeling. Indicators of LV diastolic dysfunction, an important risk factor for heart failure, calculated and used as outcomes were left atrial volume (LAV) (a marker of chronically elevated LV filling pressures), the ratio of early (E) to late (A) mitral inflow velocities (E/A), and the ratio of early (E) mitral to early (e′) myocardial velocities (E/e′) (an estimate of LV filling pressure)^33,34^.

### Pubertal timing

In NSHD, pubertal timing was indicated by stage of puberty at 1415 years in boys and by age at menarche in girls. At age 14–15 years, study members underwent examinations and interviews by school doctors which included assessments of visibility of pigmented pubic hair, visibility of axillary hair and, for boys only, development of genitalia and whether the voice had broken. Based on these indicators, boys were classified as pre-pubertal, early puberty, advanced puberty, and fully mature^17^. The fully mature group are, therefore, the group who would have experienced the earliest pubertal timing and the pre-pubertal group the latest. Age at menarche for girls was obtained from mothers’ reports. As 188 girls had not reached menarche by the date of the examination, age at menarche obtained from cohort members’ self-reports when they were aged 48 years was used where available (N = 94). Since we have previously found that recalled age at menarche showed only moderate agreement with age reported in adolescence^35^, we also categorised age at menarche into 5 groups (age 11 or under, 12, 13, 14, 15 or older which includes the group who had not reached menarche by the examination). Binary indicators distinguishing early puberty were derived: age at menarche at 11 years or under versus 12 years and over for women and the fully mature group versus the 3 less mature groups combined in men.

### Adult cardiovascular risk factors and confounders

Adult cardiovascular risk factors and confounders were collected in both the CRFs and at home visits. At age 60–64 years, height and weight were measured according to a standard protocol and BMI calculated. Brachial systolic (SBP) and diastolic blood pressure (DBP) were measured twice with the participant in a seated position using an Omron HEM-705 sphygmomanometer (OMRON UK Healthcare UK Ltd; Milton Keynes, UK); the second reading (or the first if the second was missing) was used. Overnight fasting blood samples were collected as previously described^14^. Total cholesterol, high-density lipoprotein (HDL), and triglyceride were measured with a Siemens Dimension Xpand analyser and HbA1c using the TOSOH G7 HPLC system. Information on medication use was self-reported. Cigarette smoking and leisure time physical activity (LTPA) were obtained from questionnaires; smoking behaviour was classified into current and ex-smoker/never smoker and LTPA into inactive at all ages (36, 43, 53 and 60–64) and active on at least one occasion.

Variables which have previously been shown to be associated with CVD risk and which may also influence pubertal timing were selected *a priori* as potential confounders. Height was measured at age 7, and BMI at age 7 was calculated from measured height and weight. Body size at age 7 (rather than at ages 2, 4, 6 or 11 when height and weight were also measured) was selected as age 7 is the final measurement in NSHD at which all participants are pre-pubertal. Father’s occupational social class when the cohort member was aged 4 and own occupation at 53 (manual; non-manual) were used as markers of childhood and adult SEP, respectively. Information on childhood illnesses was obtained from mothers’ reports throughout childhood. All hospital admissions experienced by study members between 0 and 15 years of age were collected through parental reports at contacts throughout childhood. Reasons for admission and length of stay were also reported. Serious childhood illness was defined as any experience of physical illness (excluding accidents) before the age of 15 years that required hospital admission of at least 28 days.

### Statistical methods

We assessed characteristics among those included and those excluded from the analysis due to missing data using t-tests for continuous variables and chi-squared tests for categorical variables. Among women, associations between age at menarche (years) and each of the markers of subclinical CVD (2 vascular measures (cIMT and PWV), 3 measures of cardiac structure (LVM, LVEDV, RWT) and 3 measures of cardiac function (LAV, E/e’, E/A) were assessed using regression models. Among men, associations with each outcome were assessed according to the 4 categories of pubertal development at 14–15 years. Models were adjusted for childhood and adult SEP and childhood serious illness.

To examine the influence of BMI, regression models were subsequently adjusted for adult BMI and height. Separate adjustment was made for BMI and height at 7 years in the subsample with these measures. All analyses were repeated with the categorical age at menarche variable and tests for a deviation from linear trend across the 5 categories were carried out, and using the binary indicators distinguishing early from later puberty.

Regression models were also used to relate pubertal timing to BMI and height and CV risk factors, and logistic regression was used for medication use, cigarette smoking and LTPA. Censored regression was used for BP, total and LDL cholesterol, triglycerides and HbA1c with individuals on antihypertensive, lipid lowering and diabetes medication, respectively, being censored at the observed value. Where there were any remaining associations between pubertal timing and any vascular or cardiac outcome after adjustment for life course SEP, childhood illness and adult BMI and height, further adjustment was made for CV risk factors identified as being associated with pubertal timing.

### Sensitivity analyses

Since the CV risk factors were also measured in those who had a home visit, we carried out a sensitivity analysis to assess whether associations were different in the larger combined CRF and home visit sample compared with the sample who visited a CRF. We also carried out a sensitivity analysis to assess the potential impact of differential mortality rates by pubertal timing on any observed associations between pubertal timing and the cardiac and vascular outcomes. We used Cox proportional hazard regression models to investigate the relationships between age at menarche in women and pubertal stage at 14–15 years in men with all-cause mortality. Study members were flagged for subsequent notification of death on the National Health Service Central Register (NHSCR) in 1971 when aged 26 years. A total of 4454 men and women alive and resident in Britain were flagged. Of those excluded, 881 had died or emigrated, and 27 could not be flagged. In Cox models, the follow-up time was from January 1971 until the date of either death or emigration or the end of March 2006 (the cohort’s 60^th^ birthday and the start of age 60–64 data collection). Follow-up time was treated as censored for those who emigrated or survived to the end of follow-up.

## Results

In the sample of 672 males and 713 females with at least one valid outcome measure and information on pubertal timing, the majority of boys were in the middle two categories of pubertal development with only 10% in the least mature group (Table 1). The median age at menarche was 13 years with 16% reaching menarche under 12 years (Table 1). Expected sex differences were observed in the cardiac and vascular measures, with men for example, having higher mean cIMT and LVM than women. The sample included in analyses was similar in terms of their outcomes to those excluded, but had slightly higher PWV, as well as higher BP and BMI (Supplementary Table 1).There was no evidence of a difference in the distribution of either age at menarche (in categories) (p = 0.2), or pubertal stage at 1415 years in boys (p = 0.5), between those included in the analytical sample and those with a measure of pubertal timing but excluded from analysis. The mean age at menarche was also 13 years in both groups.

**Table 1:** Descriptive statistics for timing of puberty, cardiac and vascular outcomes and CV risk factors at 60–64 years by sex.

	Men (maximum n = 672)				Women (maximum n = 713)			
	n	(%)	Mean	(SD)	n	(%)	Mean	(SD)
**Age at puberty**								
1 Stage 4 at 15 y (boys) or age at menarche ≤11 y (girls)	174	(25.9)			115	(16.1)		
2 Stage 3 at 15 y (boys) or age at menarche 12 y (girls)	205	(30.5)			220	(30.9)		
3 Stage 2 at 15 y (boys) or age at menarche 13 y (girls)	228	(33.9)			245	(34.4)		
4 Stage 1 at 15 y (boys) or age at menarche 14 y (girls)	65	(9.7)			99	(13.9)		
5 Age at menarche ≥15 y (girls)	—	—			34	(4.8)		
Carotid intima-media thickness (cIMT), mm	517		0.71	(0.14)	565		0.67	(0.11)
Pulse wave velocity (PWV), m/s	486		9.1	(8.4)	549		9.3	(12.5)
Left ventricular mass (LVM), g	588		209.4	(60.4)	636		155.9	(44.4)
Left ventricular end diastolic volume (LVEDV), ml	630		112.2	(30.1)	663		84.5	(19.9)
Relative wall thickness (RWT)	588		0.42	(0.09)	636		0.41	(0.08)
Left atrial volume (LAV), ml	579		43.9	(15.4)	585		35.3	(12.8)
E/A	622		1.0	(0.3)	672		1.0	(0.3)
E/e’	579		7.5	(2.0)	650		8.3	(2.0)
Taking antihypertensive medication (/total n)	150/672	(22.3)			117/713	(16.4)		
SBP, mmHg	672		139.6	(18.2)	711		133.2	(17.9)
DBP, mmHg	672		79.1	(9.7)	711		75.9	(9.5)
Taking lipid lowering medication (/total n)	177/672	(26.3)			122/712	(17.1)		
Total cholesterol, mmol/L	650		5.3	(1.1)	672		6.0	(1.2)
LDL cholesterol, mmol/L	627		3.3	(1.0)	660		3.7	(1.0)
HDL cholesterol, mmol/L	650		1.4	(0.3)	672		1.8	(0.4)
Triglycerides, mmol/L	632		1.2*	(1.2, 1.3)*	661		1.0*	(1.0, 1.1)*
Taking diabetes medication (/total n)	33/672	(4.9)			27/713	(3.8)		
HbA1c, mmol/mol	627		39.6*	(39.1, 40.0)*	661		39.6*	(39.2, 40.1)*
BMI, kg/m^2^	672		27.9	(4.0)	713		27.6	(5.2)
Height, m	672		1.75	(0.06)	713		1.62	(0.06)
Inactive at all ages (/total n)	86/672	(12.8)			106/713	(14.9)		
Current smoker (/total n)	68/663	(10.3)			75/706	(10.6)		

*Geometric mean (95% CI).

cIMT: carotid intima-media thickness; PWV: pulse wave velocity; LVM: left ventricular mass; LVEDV: left ventricular end diastolic volume; RWT: relative wall thickness; LAV: left atrial volume; SBP: systolic blood pressure; DBP: diastolic blood pressure; LDL: low density lipoprotein; HDL: high density lipoprotein; HbA1c: glycated haemoglobin; BMI: body mass index.

### Vascular outcomes

There was no evidence of an association between age at menarche and either cIMT or PWV (Table 2) or between pubertal stage and either outcome in men (Table 3). Adjustment for adult and childhood SEP and childhood illness had little effect on the associations in women (Supplementary Table 3) or men (Supplementary Table 4).

**Table 2:** Cardiac and vascular outcomes at age 60–64 y by age at menarche in women*.

	Age at menarche (per year)			Later menarche ≥12 years versus early menarche ≤11 years	
	n	Reg. Coeff. (95% CI)	p-value	Reg. Coeff. (95% CI)	p-value
cIMT, mm	538	−0.004 (−0.011, 0.004)	0.3	0.001 (−0.024, 0.026)	0.9
PWV, m/s	524	−0.29 (−1.22, 0.63)	0.5	−0.85 (−3.87, 2.17)	0.6
LVM, g	606	−4.21 (−7.04, −1.38)	0.004	−10.56 (−20.25, −0.87)	0.03
LVEDV, ml	629	−1.29 (−2.55, −0.02)	0.05	−5.66 (−9.94, −1.39)	0.01
RWT	606	−0.004 (−0.010, 0.001)	0.2	−0.008 (−0.027, 0.010)	0.4
LAV, ml	556	−0.97 (−1.81, −0.12)	0.02	−0.83 (−3.74, 2.08)	0.6
E/A	639	0.001 (−0.017, 0.019)	0.9	−0.022 (−0.083, 0.039)	0.5
E/e’	617	−0.11 (−0.24, 0.02)	0.09	−0.13 (−0.58, 0.31)	0.6

*Regression coefficients from unadjusted models in sample with complete information on confounders and adult height and BMI.

cIMT: carotid intima-media thickness; PWV: pulse wave velocity; LVM: left ventricular mass; LVEDV: left ventricular end diastolic volume; RWT: relative wall thickness; LAV: left atrial volume.

**Table 3:** Cardiac and vascular outcomes at age 60–64 y by stage of puberty at 14-15 y in men*.

	n	1 Early puberty	2	3	4 Late puberty	p-value (trend)**	Later puberty (groups 2,3,4) versus earliest puberty	
			Reg. Coeff. (95% CI)	Reg. Coeff. (95% CI)	Reg. Coeff. (95% CI)		Reg. Coeff. (95% CI)	p-value
cIMT, mm	500	ref	−0.008 (−0.040, 0.024)	−0.013 (−0.045, 0.019)	−0.023 (−0.069, 0.022)	0.3	−0.012 (−0.040, 0.015)	0.4
PWV, m/s	474	ref	1.54 (−0.47, 3.55)	0.30 (−1.72, 2.31)	−0.31 (−3.34, 2.72)	0.8	0.78 (−0.97, 2.53)	0.4
LVM, g	571	ref	−12.92 (−26.36, 0.51)	−8.03 (−21.04, 4.99)	−6.42 (−25.15, 12.32)	0.4	−9.79 (−21.24, 1.66)	0.09
LVEDV, ml	612	ref	0.91 (−5.53, 7.35)	−1.96 (−8.27, 4.35)	0.61 (−8.42, 9.65)	0.7	−0.44 (−5.97, 5.10)	0.9
RWT	571	ref	−0.013 (−0.032, 0.006)	0.004 (−0.015, 0.023)	−0.001 (−0.028, 0.026)	0.6	−0.004 (−0.020, 0.013)	0.7
LAV, ml	562	ref	0.35 (−3.06, 3.75)	−1.26 (−4.59, 2.06)	−3.09 (−7.90, 1.73)	0.2	−0.84 (−3.75, 2.07)	0.6
E/A	605	ref	−0.008 (−0.065, 0.048)	0.024 (−0.032, 0.079)	0.030 (−0.049, 0.109)	0.3	0.011 (−0.037, 0.060)	0.7
E/e′	562	ref	−0.10 (−0.56, 0.36)	−0.16 (−0.61, 0.29)	0.06 (−0.57, 0.68)	0.8	−0.11 (−0.50, 0.28)	0.6

*Regression coefficients from unadjusted models in sample with complete information on confounders and adult height and BMI.

**P for linear trend across groups.

cIMT: carotid intima-media thickness; PWV: pulse wave velocity; LVM: left ventricular mass; LVEDV: left ventricular end diastolic volume; RWT: relative wall thickness; LAV: left atrial volume.

### Cardiac outcomes

Later age at menarche per year was associated with better LV structure (i.e. lower LVM and LVEDV, and more weakly with lower RWT), and with better LV function (i.e. lower LAV and filling pressure (E/e’)) (Table 2). Similar results were found for categorical age at menarche with no evidence of deviation from linear trend for any outcome (Supplementary Table 2). The means of markers of LV structure (LVM and LVEDV) were lower (i.e. better) in the later compared with the earliest menarche group (≤11 years) (Table 2).

In men, there were no clear association across the 4 groups of stage of puberty and any measure of cardiac structure or function (Table 3). Those in the three later puberty groups had a mean LVM −9.8 g (95% CI: −21.2, 1.7) lower than the earliest group.

Adjustment for adult and childhood SEP and childhood illness had little effect on the associations in women (Supplementary Table 3) and associations remained null in men (Supplementary Table 4). The associations between age at menarche and cardiac structure and function were substantially attenuated when adult BMI and height were included in the model. However, in terms of LV structure, LVEDV and, to a lesser extent, LVM, remained higher in those reaching menarche at ≤11 years compared to those reaching it later (Table 4). Attenuation was due to addition of BMI rather than height (Supplementary Table 5). Similarly, adjustment for childhood BMI and height led to attenuation of all measures, except for LV filling pressure (E/e’) which was strengthened (Table 4) due to adjustment for height (Supplementary Table 5).

**Table 4:** Cardiac and vascular outcomes at age 60–64 y by age at menarche in women adjusting for adult BMI and height and, separately, BMI and height at age 7 y.

	Age at menarche (per year)			Later menarche ≥12 years versus early menarche ≤11 y	
	n	Reg. Coeff. (95% CI)	p-value	Reg. Coeff. (95% CI)	p-value
**Adjusted for childhood and adult SEP, childhood illness and adult BMI and height**					
cIMT, mm	538	−0.004 (−0.012, 0.003)	0.3	−0.002 (−0.027, 0.023)	0.9
PWV, m/s	524	−0.33 (−1.26, 0.60)	0.5	−0.87 (−3.88, 2.14)	0.6
LVM, g	606	−2.19 (−4.77, 0.39)	0.1	−7.56 (−16.27, 1.15)	0.09
LVEDV, ml	629	−0.53 (−1.73, 0.68)	0.4	−4.50 (−8.53, −0.53)	0.03
RWT	606	−0.002 (−0.007, 0.004)	0.5	−0.003 (−0.022, 0.016)	0.7
LAV, ml	556	−0.48 (−1.27, 0.32)	0.2	−0.24 (−2.94, 2.46)	0.9
E/A	639	−0.006 (−0.024, 0.012)	0.5	−0.029 (−0.089, 0.032)	0.4
E/e’	617	−0.08 (−0.21, 0.06)	0.3	−0.07 (−0.51, 0.37)	0.8
**Adjusted for childhood and adult SEP, childhood illness and BMI and height at age 7**					
cIMT, mm	488	−0.003 (−0.011, 0.004)	0.4	0.001 (−0.026, 0.027)	>0.99
PWV, m/s	477	−0.09 (−1.11, 0.93)	0.9	−0.42 (−3.74, 2.91)	0.8
LVM, g	551	−2.47 (−5.50, 0.56)	0.1	−7.94 (−18.11, 2.23)	0.1
LVEDV, ml	572	−0.39 (−1.75, 0.97)	0.6	−3.41 (−8.00, 1.12)	0.1
RWT	551	−0.004 (−0.010, 0.002)	0.2	−0.013 (−0.032, 0.007)	0.2
LAV, ml	505	−0.51 (−1.42, 0.40)	0.3	0.82 (−2.28, 3.93)	0.6
E/A	584	0.001 (−0.019, 0.020)	0.99	−0.019 (−0.084, 0.046)	0.6
E/e’	562	−0.18 (−0.33, −0.04)	0.01	−0.32 (−0.80, 0.15)	0.2

SEP: Socioeconomic position; cIMT: carotid intima-media thickness; PWV: pulse wave velocity; LVM: left ventricular mass; LVEDV: left ventricular end diastolic volume; RWT: relative wall thickness; LAV: left atrial volume.

### CV risk factors

In women, later menarche was associated with lower BMI, taller height and lower triglyceride levels as well as with lower odds of lipid lowering and antihypertensive medication use and, more weakly, diabetes medication use (Table 5). Further adjustments were therefore made for triglyceride levels and lipid lowering and antihypertensive medication where there was evidence of remaining associations between pubertal timing and the cardiac outcomes after adjustment for confounders and BMI and height. Associations between age at menarche ( ≤ 11 versus older) and 2 markers of cardiac structure (LVM, LVEDV) and age at menarche (years) and the marker of function (E/e′) were not further attenuated after additional adjustment for lipid lowering and antihypertensive medication and triglycerides.

**Table 5:** Established cardiovascular risk factors at 60–64 years and age of menarche (girls) or pubertal stage at 15 years (boys) in the analytic sample.

	Women			n	Men (pubertal stage at 14-15 years)				
	Age at menarche (year)				1 Early puberty	2	3	4 Late puberty	p-value (trend)
	n	Reg Coeff. (95% CI)	p-value			Reg. Coeff. (95% CI)	Reg. Coeff. (95% CI)	Reg. Coeff. (95% CI)	
BMI, kg/m^2^	713	−0.80 (−1.11, −0.49)	<0.001	672	ref	−0.77 (−1.57, 0.03)	−1.06 (−1.84, −0.28)	−0.38 (−1.50, 0.75)	0.08
Height, m	713	0.005 (0.002, 0.009)	0.002	672	ref	−0.006 (−0.019, 0.007)	−0.005 (−0.018, 0.007)	−0.015 (−0.032, 0.004)	0.2
SBP*, mmHg	711	−0.56 (−1.78, 0.66)	0.4	672	ref	−3.74 (−7.92, 0.43)	−4.55 (−8.64, −0.47)	−5.45 (−11.33, 0.43)	0.03
DBP*, mmHg	711	−0.08 (−0.72, 0.55)	0.8	672	ref	−1.58 (−3.82, 0.65)	−2.22 (−4.41, −0.03)	−1.43 (−4.58, 1.71)	0.1
Total cholesterol*, mmol/L	671	0.02 (−0.05, 0.09)	0.6	650	ref	−0.03 (−0.24, 0.19)	0.06 (−0.15, 0.28)	0.13 (−0.17, 0.44)	0.3
LDL cholesterol *, mmol/L	659	0.03 (−0.03, 0.10)	0.3	627	ref	0.03 (−0.16, 0.22)	0.07 (−0.12, 0.25)	0.07 (−0.20, 0.34)	0.5
HDL cholesterol, mmol/L	672	0.012 (−0.013, 0.037)	0.4	650	ref	−0.005 (−0.071, 0.061)	0.003 (−0.062, 0.067)	0.058 (−0.035, 0.151)	0.4
Triglycerides*, %	660	−4.41** (−7.82, −1.01)	0.01	632	ref	−5.47** (−18.20, 7.25)	1.53** (−10.95, 14.00)	2.75** (−15.21, 20.71)	0.6
HbA1c*,%	661	−0.14** (−1.06, 0.78)	0.8	627	ref	−1.47** (−4.71, 1.78)	−3.36** (−6.54, −0.19)	−2.41** (−7.00, 2.18)	0.06
Antihypertensive medication (yes vs. no)	713	0.82 (0.69, 0.97)	0.02	627	ref	0.66 (0.41, 1.06)	0.72 (0.45, 1.14)	0.59 (0.29, 1.21)	0.1
Lipid lowering medication (yes vs. no***)	712	0.78 (0.61, 0.94)	0.003	672	ref	0.56 (0.35, 0.89)	0.68 (0.44, 1.06)	0.85 (0.46, 1.58)	0.3
Diabetes medication (yes vs. no***)	713	0.78 (0.56, 1.08)	0.1	672	ref	0.40 (0.17, 0.96)	0.31 (0.13,0.78)	0.31 (0.07, 1.40)	0.01
Smoking status (ex/non-smoker vs. smoker ***)	706	0.91 (0.75, 1.10)	0.3	663	ref	1.13 (0.59, 2.17)	1.10 (0.59, 2.07)	2.68 (0.77, 9.33)	0.3
LTPA (active vs. inactive ***)	713	1.08 (0.91, 1.28)	0.4	672	ref	0.87 (0.47, 1.59)	0.94 (0.52, 1.72)	0.98 (0.41, 2.33)	0.96

*Censored regression.

**Logged outcomes.

***Odds ratio from logistic regression model.

BMI: Body Mass Index; SBP: systolic blood pressure; DBP: diastolic blood pressure; BMI: body mass index; LDL: low density lipoprotein; HDL: high density lipoprotein; HbA1c: glycated haemoglobin; LTPA: Lifetime physical activity.

Among men, later puberty was related lower SBP and lower odds of diabetes medication use and weakly related to lower BMI,DBP and HbA1c and lower odds of antihypertensive medication use (Table 5).

### Sensitivity analyses

Similar, although stronger associations, due to increased statistical power, were observed in the larger sample (Supplementary Table 6).

There was no association between age at menarche and all-cause mortality (HR (95% CI) = 1.02 (0.87,1.12) p = 0.8 per year later menarche) (N = 1670), but evidence that later maturing men had higher rates of mortality than those maturing early (HR (95% CI) = 2.41 (1.36,4.27); p = 0.003 for least mature at 15 versus fully mature) (n = 1897). Deaths from CVD were only slightly greater in the least mature group compared to the most mature, although estimates lacked precision due to the smaller number of events.

## Discussion

Our findings provide little evidence to support a direct effect of pubertal timing on markers of vascular and cardiac structure and function in early old age. The relationships between earlier age at menarche in women and more adverse cardiac structure (higher LVM and LVEDV) and function (higher LAV) were largely explained by higher adult and/or childhood BMI. The null findings seen in men are despite a persisting relationship between earlier puberty and higher blood pressure but lower all-cause mortality.

No associations between age at menarche and cIMT were observed in the Young Finns study^11^ or among white women in the Bogalusa Heart Study^13^, or in a study of German women aged 50–81^12^ consistent with our findings, as the NSHD is an ethnically homogeneous cohort being representative of those born in Britain in 1946. In Black women in the Bogalusa Heart Study, those with a very early menarche (under 11 years) had higher cIMT than those with later menarche^13,12^. In the German study, late menarche (>15 years) was found to be associated with lower odds of peripheral artery disease measured using ankle-brachial index compared with menarche between 12–15 years^36^. To our knowledge there are no previous studies investigating pubertal timing and cardiac measures in general population samples, but one small study found that girls with precocious puberty had higher LVM than age-matched girls with puberty within the normal range, even after adjusting for total body fat^37^. However, the mechanism underlying the effect in this extreme group is likely to be different to any within the normal range of puberty.

We found that the associations between earlier menarche and more adverse levels of two markers of LV structure (LVM, LVED) and a marker of chronically elevated filling pressure (LAV) were at least partially explained by increased BMI in childhood and adulthood. Gains in adiposity in childhood are associated with greater cardiac growth^38^, and we have previously observed in NSHD that there remained an association between childhood BMI and cardiac structure in adulthood which was not entirely mediated through current body size^39^. Left ventricular size scales in proportion to body size, but the appropriate mode of indexation remains contentious. Since pubertal timing is related to growth and adult body size, we chose not to index the measures of mass or volume, but to include adult height and BMI as covariates in models. Since women who reach menarche earlier tend to be shorter in adulthood, while those of shorter height have smaller hearts, addition of adult height strengthened the observed associations, and thus the attenuation was due to adult BMI.

Our findings are consistent with two Mendelian randomisation studies, using a genetic risk score as an instrumental variable for pubertal timing and which, unlike observational studies, are not subject to confounding. The first suggested a lack of causal association between age at menarche and most conventional CV risk factors, concluding that observational studies showing associations may be confounded by factors such as childhood obesity^40^. Similarly, the second such study also suggested little evidence of an effect of pubertal timing in either sex on cardiometabolic outcomes concluding that associations are largely confounded by pre-pubertal adiposity^41^. Our findings show an attenuation of associations after adjustment for childhood BMI supporting this conclusion. Further, since there were low rates of childhood overweight in NSHD compared with later-born British cohorts^42^, there is likely to be less confounding by childhood obesity in our study compared to others which show stronger relationships and cannot adjust for early life adiposity.

Our results are not necessarily inconsistent with findings from large studies with disease outcomes and greater statistical power to assess the extremes of the age at menarche distribution. Both the Million Women’s Study^3^ and the UK Biobank Study^4^ observed greater risk of self-reported CV-related outcomes in those with very early menarche (<11 years and 8–11 years, respectively); perhaps consistent with our findings of increased LVEDV, and possibly LVM, in those reaching menarche at ≤11 years even after adjustment for adult BMI. Different mechanisms may be acting at the extremes of the distribution of age at menarche compared with the normal range.

It is possible that true associations in our study could have been weakened in the analytical sample, due to those attending the CRF at 60–64 y being healthier than others^29^. However, similar associations were seen for BP in men and triglycerides in women to those previously observed at age 53 y in NSHD in a larger and less selective sample^14,17^. Differential mortality rates by pubertal timing are unlikely to have biased the findings as there was no evidence of an association between age at menarche and mortality in women, while it was later, rather than earlier, maturing boys who had higher all-cause, although not CVD, mortality rates. These findings suggest the need to further investigate effects of puberty on CV risk and other health outcomes in younger men. For example, later puberty was associated with increased risk of adolescent onset affective symptoms in NSHD^43^, and the UK Biobank Study found later age at voice breaking to be related to increased likelihood of depression^4^.

To our knowledge, no other study has both prospectively collected information on pubertal timing in males as well as females, and detailed objective measures of cardiac and vascular structure and function in older age. The prospectively measured pubertal timing is a key strength, as only moderate agreement has been found between age at menarche recalled in midlife and recorded in adolescence^35^, meaning that the pubertal measures are not subject to recall bias. The measures of cardiac structure and function allow consideration of objective measures of pre-clinical disease rather than self-reported disease. The ability to adjust for measured pre-pubertal body size, unlike many previous studies, means we are able to adjust for confounding by childhood adiposity. There are also a number of weaknesses. Our study has limited power to detect possible non-linear associations, and in particular to study very early (≤10 years) and very late puberty (≥17); small groups found to be at most risk of disease in previous studies. Missing data are inevitable in long-running studies such as the NSHD, and the sample included in analyses are healthier and of more advantaged SEP than the original cohort. However, they remain representative in terms of pubertal timing and our sensitivity analyses suggested that mortality and drop-out since age 53 years are unlikely to have had a major influence on the findings. The NSHD study members were born in the early post-war period, with lower rates of childhood obesity than more recent generations, and therefore our findings may not be generalizable to later-born cohorts. Given that we selected 8 primary outcomes and men and women were tested separately, spurious positive findings are a possibility. However, our findings were consistent across groups of variables and by sex and our overall conclusion is that there is little evidence of any association.

## Conclusions

Our findings suggest that any effect of pubertal timing within the normal range on cardiac and vascular structure and function is likely to be small and be primarily confounded by pre-pubertal BMI and/or mediated through adult adiposity. Links between late maturation and increased mortality but lower BP in men require further investigation.

## Supplementary information

Supplementary Materials

## Data Availability

Data used in this publication are available to bona fide researchers upon request to the NSHD Data Sharing Committee via a standard application procedure. Further details can be found at http://www.nshd.mrc.ac.uk/data. doi:10.5522/NSHD/Q101; 10.5522/NSHD/Q102.
