# Supplementary material for: The relationship between pubertal timing and markers of vascular and cardiac structure and function in men and women aged 60–64 years
Source: Sci Rep. 2019 Jul 30;9:11037. doi: 10.1038/s41598-019-47164-x (PMC6667431; doi:10.1038/s41598-019-47164-x)
Supplement: Supplementary file 1 — Supplementary Materials [file 41598_2019_47164_MOESM1_ESM.docx]

**Pubertal timing and cardiovascular ageing in men and women: supplementary material**

| **Supplementary table 1**. Comparison of excluded versus included participants (out of those attending the clinic visit at 60-64 years) | | | | | | | | | | | |
| --- | --- | --- | --- | --- | --- | --- | --- | --- | --- | --- | --- |
|  |  | Excluded (n=305) | | | |  | Included (n=1385) | | | |  |
|  |  | n | (%) | Mean | (SD) |  | n | (%) | Mean | (SD) | P-value** |
| Sex |  |  |  |  |  |  |  |  |  |  |  |
|  | *Male* | 141 | (46.2) |  |  |  | 672 | (48.5) |  |  | 0.5 |
|  | *Female* | 164 | (53.8) |  |  |  | 713 | (51.5) |  |  |  |
| cIMT, mm | | 247 |  | 0.68 | (0.13) |  | 1,082 |  | 0.69 | (0.12) | 0.7 |
| PWV, m/s | | 255 |  | 8.47 | (5.19) |  | 1,035 |  | 9.20 | (10.77) | 0.04 |
| LVM, g | | 260 |  | 179.5 | (60.6) |  | 1,224 |  | 181.6 | (59.1) | 0.5 |
| LVEDV, ml | | 281 |  | 99.0 | (28.7) |  | 1,293 |  | 98.0 | (28.9) | 0.5 |
| RWT | | 260 |  | 0.41 | (0.09) |  | 1,224 |  | 0.42 | (0.09) | 0.5 |
| LAV, ml | | 249 |  | 40.7 | (16.1) |  | 1,164 |  | 39.6 | (14.8) | 0.3 |
| E/A | | 289 |  | 1.02 | (0.29) |  | 1,294 |  | 0.99 | (0.27) | 0.2 |
| E/e’ | | 267 |  | 7.84 | (2.21) |  | 1,229 |  | 7.93 | (2.07) | 0.4 |
| Taking antihypertensive medication | | 56 | (18.4) |  |  |  | 267 | (19.3) |  |  | 0.7 |
| SBP, mmHg | | 305 |  | 133.4 | (17.3) |  | 1,383 |  | 136.29 | (18.3) | 0.01 |
| DBP, mmHg | | 305 |  | 76.3 | (10.1) |  | 1,383 |  | 77.4 | (9.7) | 0.04 |
| Taking lipid lowering medication | | 58 | (19.0) |  |  |  | 299 | (21.6) |  |  | 0.3 |
| Total cholesterol, mmol/L | | 293 |  | 5.8 | (1.2) |  | 1,322 |  | 5.7 | (1.2) | 0.2 |
| LDL cholesterol, mmol/L | | 283 |  | 3.6 | (1.0) |  | 1,287 |  | 3.5 | (1.0) | 0.3 |
| HDL cholesterol, mmol/L | | 293 |  | 1.6 | (0.4) |  | 1,322 |  | 1.6 | (0.4) | 0.1 |
| Triglycerides, mmol/L | | 285 |  | 1.1* | (1.0, 1.1)* |  | 1,293 |  | 1.1* | (1.1, 1.2)* | 0.1 |
| Taking diabetes medication | | 12 | (3.9) |  |  |  | 60 | (4.3) |  |  |  |
| HbA1c, mmol/mol | | 289 |  | 39.7* | (39.0,40.4)* |  | 1,288 |  | 39.6* | (39.3, 39.9)* | 0.9 |
| BMI, kg/m^2^ | | 305 |  | 27.2 | (4.6) |  | 1,385 |  | 27.8 | (4.7) | 0.04 |
| Height, m | | 305 |  | 1.69 | (0.09) |  | 1,385 |  | 1.7 | (0.1) | 0.6 |
| Inactive at all ages | | 41 | (13.5) |  |  |  | 192 | (13.9) |  |  | 0.9 |
| Current smoker | | 30 | (10.1) |  |  |  | 143 | (10.5) |  |  | 0.8 |
| *geometric mean (95% CI)  **p-value from chi square test for categorical variables or t-test/Wilcoxon rank sum for continuous variables  cIMT: carotid intima-media thickness; PWV: pulse wave velocity; LVM: left ventricular mass; LVEDV: left ventricular end diastolic volume; RWT: relative wall thickness; LAV: left atrial volume; SBP: systolic blood pressure; DBP: diastolic blood pressure; LDL: low density lipoprotein; HDL: high density lipoprotein; HbA1c: glycated haemoglobin; BMI: body mass index | | | | | | | | | | | |

| **Supplementary table 2.** Cardiac and vascular outcomes at age 60-64y by age at menarche in women* | | | | | | | | | |
| --- | --- | --- | --- | --- | --- | --- | --- | --- | --- |
|  |  | **≤11y years** | **12 years** | **13 years** | **14 years** | | **≥15 years** |  | |
|  | n |  | Reg. Coeff.  (95% CI) | Reg. Coeff.  (95% CI) | Reg. Coeff.  (95% CI) | Reg. Coeff.  (95% CI) | | P-value (trend)** | |
| cIMT, mm | 538 | ref | 0.009  (-0.020, 0.037) | -0.002  (-0.030, 0.025) | -0.003  (-0.035, 0.030) | | -0.014  (-0.060, 0.044) | | 0.4 |
| PWV, m/s | 524 | ref | -0.56  (-3.99, 2.88) | -0.53  (-3.84, 2.79) | -1.89  (-5.95, 2.17) | | -2.33  (-8.26, 4.05) | | 0.3 |
|  |  |  |  |  |  | |  | |  |
| LVM, g | 606 | ref | -9.80  (-20.69, 1.10) | -9.19  (-19.87, 1.49) | -9.25  (-22.09, 3.59) | | -28.63  (-46.65, 8.77) | | 0.02 |
| LVEDV, ml | 629 | ref | -5.51  (-10.33, -0.69) | -4.80  (-9.52, -0.07) | -7.59  (-13.34, -1.84) | | -7.44  (-15.46, 0.43) | | 0.02 |
| RWT | 606 | ref | -0.004  (-0.025, 0.017) | -0.012  (-0.033, 0.009) | 0.001  (-0.024, 0.026) | | -0.031  (-0.067, 0.036) | | 0.2 |
| LAV, ml | 556 | ref | -0.40  (-3.70, 2.90) | 0.11  (-3.10, 3.32) | -2.16  (-5.99, 1.68) | | -5.93  (-11.35, 3.26) | | 0.07 |
| E/A | 639 | ref | -0.021  (-0.090, 0.048) | -0.018  (-0.085, 0.050) | -0.049  (-0.131, 0.033) | | 0.018  (-0.098, 0.066) | | 0.6 |
| E/e’ | 617 | ref | -0.18  (-0.67,0.32) | 0.11  (-0.37, 0.60) | -0.38  (-0.97, 0.21) | | -0.92  (-1.76, 0.45) | | 0.1 |
| * regression coefficients from unadjusted models in sample with complete information on confounders and adult height and BMI  **no evidence of deviation from linearity in any analyses  cIMT: carotid intima-media thickness; PWV: pulse wave velocity; LVM: left ventricular mass; LVEDV: left ventricular end diastolic volume; RWT: relative wall thickness; LAV: left atrial volume | | | | | | | | | |

| **Supplementary table 3.** Cardiac and vascular outcomes at age 60-64y by age at menarche in women adjusted for childhood and adulthood socioeconomic position and childhood illness with complete information on adult height and BMI | | | | | | |
| --- | --- | --- | --- | --- | --- | --- |
|  | **Age at menarche (per year)** | | |  | **Later menarche ≥12 years**  **versus early menarche ≤11y** | |
|  | n | Reg. Coeff.  (95% CI) | p-value |  | Reg. Coeff.  (95% CI) | p-value |
| cIMT, mm | 538 | -0.004  (-0.011, 0.004) | 0.3 |  | -0.001  (-0.026, 0.024) | 0.9 |
| PWV, m/s | 524 | -0.26  (-1.17, 0.65) | 0.6 |  | -0.78  (-3.77, 2.21) | 0.6 |
|  |  |  |  |  |  |  |
| LVM, g | 606 | -4.45  (-7.28, -1.62) | 0.002 |  | -12.07  (-21.79, -2.34) | 0.02 |
| LVEDV, ml | 629 | -1.32  (-2.59, -0.06) | 0.04 |  | -5.90  (-10.20, -1.61) | 0.01 |
| RWT | 606 | -0.004  (-0.010, 0.001) | 0.1 |  | -0.009  (-0.027, 0.010) | 0.4 |
| LAV, ml | 556 | -0.97  (-1.82, -0.13) | 0.02 |  | -0.88  (-3.81, 2.04) | 0.6 |
| E/A | 639 | 0.003  (-0.015, 0.021) | 0.8 |  | -0.01  (-0.070, 0.052) | 0.8 |
| E/e’ | 617 | -0.12  (-0.25, 0.01) | 0.08 |  | -0.18  (-0.63,0.26) | 0.4 |
| cIMT: carotid intima-media thickness; PWV: pulse wave velocity; LVM: left ventricular mass; LVEDV: left ventricular end diastolic volume; RWT: relative wall thickness; LAV: left atrial volume | | | | | | |

| **Supplementary table 4.** Cardiac and vascular outcomes at age 60-64y by stage of puberty at 15y in men adjusted for childhood and adulthood socioeconomic position and childhood illness with complete information on adult height and BMI | | | | | | | | | | |
| --- | --- | --- | --- | --- | --- | --- | --- | --- | --- | --- |
|  |  | **1**  **Early puberty** | **2** | **3** | **4**  **Late puberty** |  |  |  | **Later puberty (2,3,4)**  **versus earliest puberty** | |
|  | n |  | Reg. Coeff.  (95% CI) | Reg. Coeff.  (95% CI) | Reg. Coeff.  (95% CI) | p-value (trend)* |  |  | Reg. Coeff.  (95% CI) | p-value |
| cIMT, mm | 500 | Ref | -0.009  (-0.041, 0.024) | -0.013  (-0.045, 0.018) | -0.023  (-0.069, 0.023) | 0.3 |  |  | -0.013  (-0.040, 0.015) | 0.4 |
| PWV, m/s | 474 | Ref | 1.59  (-0.43, 3.61) | 0.28  (-1.73, 2.30) | -0.31  (-3.35, 2.73) | 0.8 |  |  | 0.79  (-0.96, 2.55) | 0.4 |
|  |  |  |  |  |  |  |  |  |  |  |
| LVM, g | 571 | Ref | -12.94  (-26.39, 0.52) | -8.14  (-21.17, 4.89) | -6.19  (-24.94, 12.55) | 0.4 |  |  | -9.82  (-21.31, 1.65) | 0.09 |
| LVEDV, ml | 612 | Ref | 0.71  (-5.74, 7.16) | -1.89  (-8.21, 4.43) | 0.45  (-8.58, 9.49) | 0.7 |  |  | -0.51  (-6.04, 5.03) | 0.9 |
| RWT | 571 | Ref | -0.013  (-0.032, 0.006) | 0.004  (-0.015, 0.023) | -0.001  (-0.028, 0.026) | 0.6 |  |  | -0.003  (-0.020, 0.013) | 0.7 |
| LAV, ml | 562 | Ref | 0.51  (-2.90, 3.92) | -1.16  (-4.48, 2.16) | -2.98  (-7.80, 1.83) | 0.2 |  |  | -0.71  (-3.63, 2.20) | 0.6 |
| E/A | 605 | Ref | -0.01  (-0.06, 0.05) | 0.02  (-0.03, 0.08) | 0.03  (-0.05, 0.11) | 0.3 |  |  | 0.01  (-0.04, 0.06) | 0.6 |
| E/e’ | 562 | Ref | -0.09  (-0.55, 0.36) | -0.17  (-0.61, 0.28) | 0.05  (-0.57, 0.68) | 0.8 |  |  | -0.11  (-0.50, 0.28) | 0.6 |
| * *P* for linear trend across groups  cIMT: carotid intima-media thickness; PWV: pulse wave velocity; LVM: left ventricular mass; LVEDV: left ventricular end diastolic volume; RWT: relative wall thickness; LAV: left atrial volume | | | | | | | | | | |

| **Supplementary table 5.**  Cardiac and vascular outcomes at age 60-64y by age at menarche in women –models adjusted for childhood and adult socioeconomic position, childhood illness and BMI or height in a) adulthood and B) childhood | | | | | | |
| --- | --- | --- | --- | --- | --- | --- |
|  | **Age at menarche (per year) adjusted for BMI** | | |  | **Age at menarche (per year) adjusted for height** | |
|  | n | Reg. Coeff. (95% CI) | p-value |  | Reg. Coeff. (95% CI) | p-value |
| 1. **Adulthood** | | | | | | |
| cIMT, mm | 538 | -0.003  (-0.010, 0.005) | 0.5 |  | -0.004  (-0.012, 0.003) | 0.2 |
| PWV, m/s | 524 | -0.33  (-1.26, 0.60) | 0.5 |  | -0.26  (-1.18, 0.66) | 0.6 |
| LVM, g | 606 | -1.43  (-4.04, 1.19) | 0.3 |  | -5.20  (-8.00, -2.39) | <0.001 |
| LVEDV, ml | 629 | -0.20  (-1.42, 1.01) | 0.7 |  | -1.65  (-2.90, -0.39) | 0.01 |
| RWT | 606 | -0.002  (-0.008, 0.003) | 0.4 |  | -0.004  (-0.009, 0.002) | 0.2 |
| LAV, ml | 556 | -0.25  (-1.05, 0.55) | 0.5 |  | -1.20  (-2.04, -0.35) | 0.01 |
| E/A | 639 | -0.005  (-0.023, 0.013) | 0.6 |  | 0.002  (-0.016, 0.020) | 0.8 |
| E/e’ | 617 | -0.10  (-0.23, 0.04) | 0.2 |  | -0.10  (-0.23, 0.03) | 0.2 |
| 1. **Childhood** | | | | | | |
| cIMT, mm | 488 | -0.003  (-0.011, 0.005) | 0.4 |  | -0.003  (-0.010, 0.005) | 0.5 |
| PWV, m/s | 477 | -0.02  (-1.03, 0.99) | 0.97 |  | -0.32  (-1.33, 0.70) | 0.5 |
| LVM, g | 551 | -3.57  (-6.58, -0.55) | 0.02 |  | -3.26  (-6.24, -0.27) | 0.04 |
| LVEDV, ml | 572 | -0.91  (-2.27, -0.45) | 0.19 |  | -0.77  (-2.11, 0.58) | 0.3 |
| RWT | 551 | -0.004  (-0.010, 0.002) | 0.2 |  | -0.004  (-0.010, 0.002) | 0.2 |
| LAV, ml | 505 | -0.81  (-1.71, 0.10) | 0.1 |  | -0.61  (-1.51, 0.28) | 0.2 |
| E/A | 584 | 0.0005  (-0.019, 0.020) | 0.96 |  | -0.001  (-0.021, 0.018) | 0.9 |
| E/e’ | 562 | -0.13  (-0.28, 0.01) | 0.06 |  | -0.17  (-0.31, -0.03) | 0.02 |
| BMI: body mass index; cIMT: carotid intima-media thickness; PWV: pulse wave velocity; LVM: left ventricular mass; LVEDV: left ventricular end diastolic volume; RWT: relative wall thickness; LAV: left atrial volume | | | | | | |

| **Supplementary table 6.** Established cardiovascular risk factors at 60-64 years and age of menarche (females) or pubertal stage at 15 years (males) in unrestricted sample | | | | | | | | | | |
| --- | --- | --- | --- | --- | --- | --- | --- | --- | --- | --- |
|  | **Women** | | |  | **Men** | | | | | |
|  | **Age at menarche** | | |  | **Pubertal stage at 15 years** | | | | | |
|  | **(per year)** | | |  |  | **1**  **Early puberty** | **2** | **3** | **4**  **Late puberty** |  |
|  | n | Reg Coeff.  (95% CI) | p-value |  | n |  | Reg Coeff.  (95% CI) | Reg Coeff.  (95% CI) | Reg Coeff.  (95% CI) | p-value (trend) |
| BMI, Kg/m^2^ | 926 | -0.84  (-1.13, -0.54) | <0.001 |  | 882 | ref | -0.73  (-1.46, -0.01) | -0.92  (-1.62, -0.21) | -0.53  (-1.53, 0.47) | 0.06 |
| Height, m | 927 | 0.059  (0.003, 0.009) | <0.001 |  | 883 | ref | -0.003  (-0.014, 0.009) | -0.002  (-0.014, 0.009) | -0.014  (-0.030, 0.002) | 0.2 |
| Systolic blood pressure *, mmHg | 919 | -0.21  (-1.30, 0.87) | 0.7 |  | 887 | ref | -3.24  (-7.02, 0.55) | -3.95  (-7.64, -0.25) | -5.02  (-10.21, 0.16) | 0.03 |
| Diastolic blood pressure*, mmHg | 919 | 0.06  (-0.51, 0.63) | 0.8 |  | 887 | ref | -1.69  (-3.71, 0.33) | -2.02  (-3.99, -0.04) | -2.02  (-4.86, 0.67) | 0.06 |
| Total cholesterol*, mmol/L | 844 | -0.001  (-0.063, 0.062) | 0.98 |  | 837 | ref | 0.009  (-0.181, 0.199) | 0.082  (-0.104, 0.268) | 0.102  (-0.162, 0.367) | 0.3 |
| LDL cholesterol *, mmol/L | 812 | 0.004  (-0.055, 0.062) | 0.9 |  | 795 | ref | 0.088  (-0.08 0.257) | 0.099  (-0.066, 0.265) | 0.016  (-0.217, 0.250) | 0.6 |
| HDL cholesterol, mmol/L | 845 | 0.021  (-0.001, 0.044) | 0.1 |  | 837 | ref | -0.053  (-0.114, 0.009) | -0.021  (-0.081, 0.040) | 0.021  (-0.065, 0.106) | 0.8 |
| Triglycerides*, % | 815 | -5.54  (-8.79, -2.29) | 0.001 |  | 802 | ref | -1.88  (-13.70, 9.95) | 0.89  (-10.72, 12.49) | 5.25  (-11.04, 21.54) | 0.5 |
| HbA1c*, % | 838 | -0.32**  (-1.18 0.54) | 0.5 |  | 822 | ref | 0.32**  (-2.89, 3.53) | -0.11**  (-4.26, 2.02) | -2.14**  (-6.56, 2.27) | 0.2 |
| Antihypertensive medication (yes vs. no***) | 1,086 | 0.91  (0.80, 1.02) | 0.1 |  | 1063 | ref | 0.86  (0.59, 1.24) | 0.78  (0.54, 1.11) | 0.58  (0.33, 1.01) | 0.04 |
| Lipid lowering medication (yes vs. no***) | 928 | 0.79  (0.65, 0.93) | 0.001 |  | 888 | ref | 0.77  (0.52, 1.14) | 0.78  (0.54, 1.15) | 0.84  (0.49, 1.43) | 0.3 |
| Diabetes medication (yes vs. no***) | 1724 | 0.74  (0.58 0.94) | 0.02 |  | 1983 | ref | 0.59  (0.33, 1.05) | 0.60  (0.35, 1.05) | 0.32  (0.11, 0.91) | 0.02 |
| Smoking (ex/never vs. smoker***) | 1240 | 0.92  (0.82, 1.03) | 0.1 |  | 1281 | ref | 1.27  (0.86, 1.85) | 1.18  (0.82, 1.69) | 1.67  (0.95, 2.94) | 0.1 |
| LTPA (active vs. inactive***) | 1408 | 1.02  (0.93, 1.12) | 0.6 |  | 1498 | ref | 0.68  (0.48, 0.96) | 0.89  (0.62, 1.26) | 0.97  (0.59, 1.58) | 0.9 |
| *censored regression  ** Logged outcomes.  *** Odds ratio from logistic regression model  BMI: Body Mass Index; SBP: systolic blood pressure; DBP: diastolic blood pressure; BMI: body mass index; LDL: low density lipoprotein; HDL: high density lipoprotein; HbA1c: glycated haemoglobin; LTPA: Lifetime physical activity | | | | | | | | | | |
